# Selection on codon bias in yeast: a transcriptional hypothesis

**DOI:** 10.1093/nar/gkt740

**Published:** 2013-08-13

**Authors:** Edoardo Trotta

**Affiliations:** Institute of Translational Pharmacology, Consiglio Nazionale delle Ricerche (CNR), Roma 00133, Italy

## Abstract

Codons that code for the same amino acid are often used with unequal frequencies. This phenomenon is termed codon bias. Here, we report a computational analysis of codon bias in yeast using experimental and theoretical genome-wide data. We show that the most used codons in highly expressed genes can be predicted by mRNA structural data and that the codon choice at each synonymous site within an mRNA is not random with respect to the local secondary structure. Because we also found that the folding stability of intron sequences is strongly correlated with codon bias and mRNA level, our results suggest that codon bias is linked to mRNA folding structure through a mechanism that, at least partially, operates before pre-mRNA splicing. Consistent with this, we report evidence supporting the adaptation of the tRNA pool to the codon profile of the most expressed genes rather than vice versa. We show that the correlation of codon usage with the gene expression level also includes the stop codons that are normally not decoded by aminoacyl-tRNAs. The results reported here are consistent with a role for transcriptional forces in driving codon usage bias via a mechanism that improves gene expression by optimizing mRNA folding structures.

## INTRODUCTION

The DNA genomic regions that code for proteins are sequences of codons. Each codon is formed by three nucleotides and codes for one amino acid. Because 61 codons code for 20 amino acids, with the exception of methionine and tryptophan, all amino acids are coded by more than one distinct codon (two to six codons). The result is that the same protein can be coded by a high number of different DNA sequences. For example, 2 × 10^13^ different coding sequences can code for the ribosomal RPL41B protein, which is one of the smallest yeast proteins, with only 26 amino acids. Did evolution seize the opportunity to use different DNA sequences for coding a protein? Is it possible that under selective pressure, a finer genetic code, also shaping the efficiency or accuracy of gene expression, evolved? This appears to be the case because codons that code for the same amino acid, also called synonymous codons, are not used randomly and, for a wide variety of organisms, the shift from their equal use is correlated with the level of gene expression (1–4). The unequal use of synonymous codons is termed codon usage bias or codon bias. Although the exact mechanisms that generate codon bias are poorly understood, a general theory of its causes, known as the mutation-selection-drift balance model, has been commonly acknowledged (5–7). This theory assumes that the high frequency of optimal synonymous codons is maintained by selection, whereas neutral mutational pressure and genetic drift allow the minor codons to maintain their low frequency. From a mechanistic perspective, we can distinguish two main classes of possible causes of codon bias: neutral forces producing homogeneous pressure across the whole genome and selective forces acting on genes in various ways. Neutral forces are mostly attributed to mutational biases that generally determine the guanine-cytosine content (GC-content) of a genome (7–9), even though other causes has also been invoked to explain genome-wide compositional biases (10). Conversely, selective forces originate from natural selection and act differently on genes to provide a fitness advantage. It is commonly believed that selection on codon bias acts principally at the translational level: codons with more abundant cognate tRNA are translated more efficiently and correctly because they reduce ribosome pausing during elongation and decrease the probability of incorporating incorrect amino acids (11–13). The coevolution of the tRNA pool and the codon usage of highly expressed genes is a major argument invoked to explain the translational selection hypothesis (4,14,15). However, to our knowledge, no definitive evidence has been reported against a model in which the tRNA pool is adapted to codon usage rather than vice versa, or against a reciprocal tuning. In this work, we test the hypothesis that the origin of selection on codon bias could involve mechanisms that act at pre-translational levels. Our results lead to the conclusion that transcriptional processes may play a significant role in the codon choice and in the degree of codon bias of highly expressed genes. We propose a model in which, at least partially, codon bias of highly transcribed genes is the result of transcriptional and translational forces that act to improve gene expression by optimizing mRNA folding structures. The transcriptional and translational explanations of codon bias are not mutually exclusive, but the two processes can cooperate, consistent with the findings that both processes are modulated by mRNA secondary structures (16–18).

## MATERIALS AND METHODS

### Genome-wide data sets

Sequence and annotation data sets for the *Saccharomyces cerevisiae* genome (release R64) were downloaded from the Saccharomyces Genome Database website (http://www.yeastgenome.org/). Intron sequences were downloaded from the Ares lab Intron Database (version 3.0) (http://compbio.soe.ucsc.edu/yeast_introns.html). From literature sources, we obtained data sets for the transcriptome (19–22), protein abundance (23), the average values of ΔG for mRNA/DNA and DNA/DNA duplex stabilities of CDS (window size 9 bp) (24), nucleosome occupancy (25), protein and mRNA high-confidence cellular levels of 408 genes (26) and parallel analysis of RNA structure (PARS) scores (27).

### Software used to extract, process, simulate and analyze data sets

The statistical analysis was performed using standard parametric and non-parametric tests included in the Statistical package (version 8.0, Statsoft, Inc.). The minimum folding ΔG° for introns was predicted using Quikfold from the Mfold web server (http://mfold.rna.albany.edu/?q=DINAMelt/Quickfold) (28) with folding energy rules RNA version 3.0 and default parameters. CodonW (version 1.4.4) (http://codonw.sourceforge.net/) (29) was used to calculate codon bias indices: Codon Adaptation Index (CAI), Frequency of optimal codons (Fop), Effective numbers of codons (Nc) and Codon Bias Index (CBI). Simulated sequences with specific codon usage or nucleotide content were generated using GenRGenS software (version 2.0) (30). Three-base periodicity index (Pi) was computed using the formula elsewhere reported in detail (26). In general, to extract, process, simulate and analyze data and sequences, we used software developed in our laboratory in the C# language that was tested by independent computational tools and manual calculations. Our software includes programs to random shuffle sequences and data using the Fisher–Yates algorithm (31).

### Relative contributions of selective and mutational forces to the global codon usage frequency

We calculated the approximate contributions of selective and mutational forces to the global codon usage frequency (CUF) by considering codon bias as being due to a linear combination of the two forces. We considered codon bias to be 100% determined by mutation when it is equivalent to that of lowly transcribed genes and 100% determined by selection when the fraction of major codons is 1. The relative contributions were estimated using the codon fractions of the 2-fold degenerated synonymous sets:

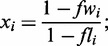

where *x_i_* is the fractional contribution of mutational bias to the global CUF estimated by the ith 2-fold degenerated family; *fw_i_* and *fl_i_* are the fractions of the major codon in the whole genome and in lowly transcribed genes, respectively. The fractional contribution of mutational bias was estimated by the average value obtained from the nine 2-fold degenerate families.

### Codon complementarity

The codon complementarity index of a transcript was calculated by dividing the number of complementary couples of codons by the total number of codons. For example, if a gene is composed by 10 AAT, 20 ATT and 100 CGA, then the codon complementarity index for that gene is 10/(10 + 20 + 100) = 0.077 because there are 10 AAT/ATT pairs.

### Comparison of the frequency variation of synonymous codons with different gene properties

We compared the variation of the use of synonymous codons associated with varying gene properties. For each data set, we first eliminated the possible outliers by removing the 0.25% of data with the highest and lowest values. Then, we group data into 20 equally spaced bins and then calculated the relative codon frequencies of each synonymous set in the bins. For each couple of properties and for each codon, we compared the relative frequency distribution in the 20 bins by calculating Pearson’s correlation coefficient (R_p_).

## RESULTS

### Relative contributions of natural selection and mutational biases in shaping the codon bias of yeast

Codon bias is the combined result of various forces with different origins. The mutation-selection-drift balance model (6,7,12) proposes two major categories for the mechanistic causes of codon bias: mutational biases and natural selection. In general, the mutational forces exert an equal pressure over all genes, thereby changing the nucleotide balance uniformly in the whole genome. Selective forces affect distinct coding sequences differently to improve fitness. Therefore, a comparative analysis of the correlation of codon usage with gene expression levels and GC-content should help to distinguish the relative contributions of selection and mutation in shaping codon bias.

In yeast, the highest correlation between codon usage and the different phases of gene expression is observed with the transcript level (26). Thus, to study the contribution of natural selection to the shaping of codon bias, we used a transcriptome data set of 6472 genes from a published source (22). The transcription levels taken from these data present an approximately log-normal frequency distribution and exhibit a suitably high level of correlation with three other independent sources of transcriptome data (0.78 < R_s_ < 0.82, 0.66 < R^2 ^< 0.69, 5061 < *N* < 5244, *P* < 10^−^^6^) (19–21).

**Figure 1.**
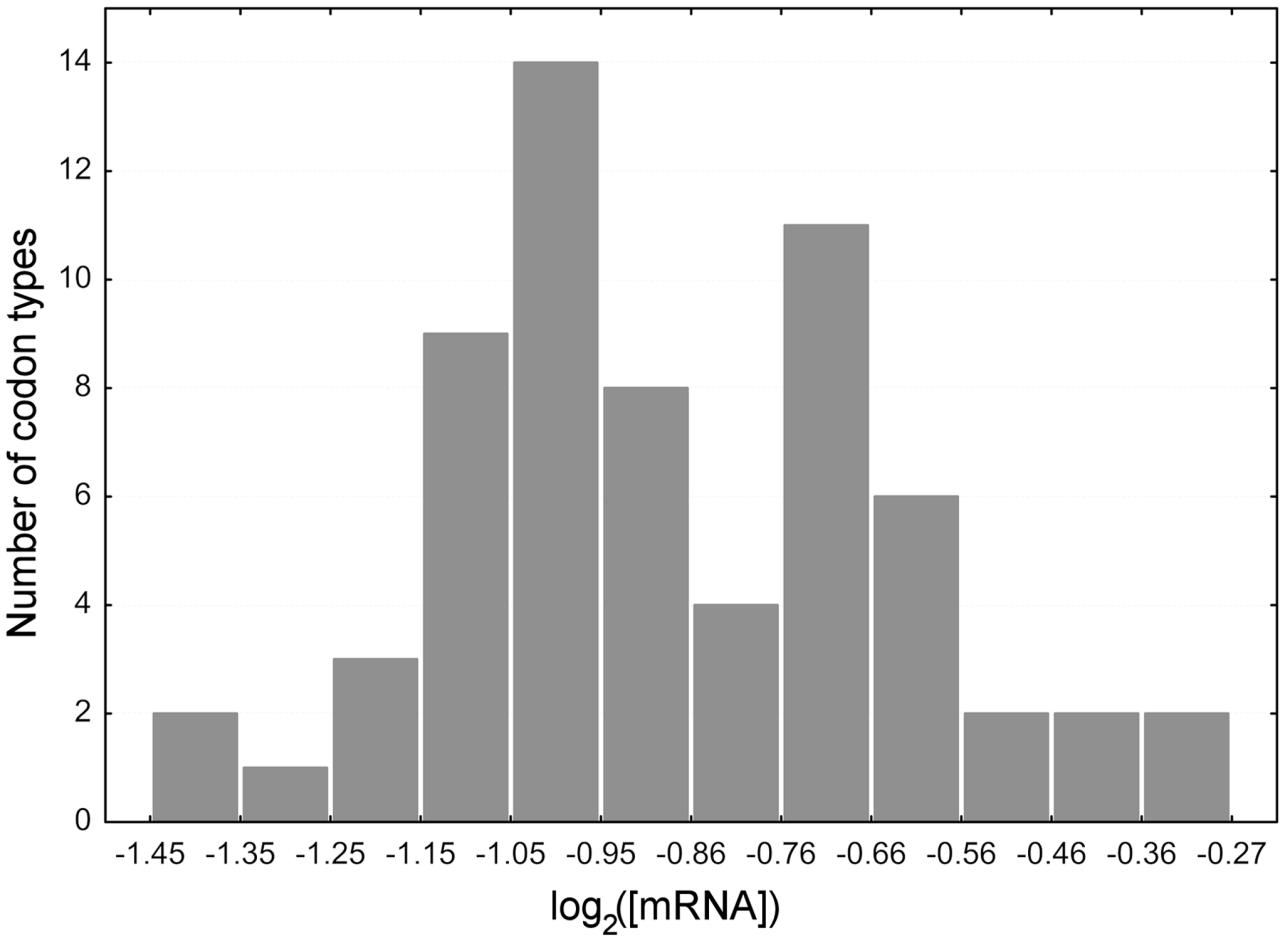
Frequency distribution of the transcript level of the 64 codon types. The average transcript levels of codons were calculated from the transcriptome data set of 6472 genes used in this work. The transcript concentration is expressed in molecule per cell.

We begin our study by estimating the average transcription level associated with all the 64 codons by assigning to each codon the level (log_2_-transformed) of the transcript in which it is found. The frequency distribution of the transcription levels for the 64 codons is bimodal (Figure 1), suggesting the presence of two distinct populations of codons associated with strongly and weakly transcribed genes. The bimodal shape is lost if transcription associated with codons are calculated after a random shuffling of the mRNA levels attributed to genes. Moreover, we back-calculated the relative transcription level of all genes from the above estimated average transcription level associated with codons and compared the results with the original experimental data. Back-calculation was performed by assigning at each gene the average value of the estimated transcription level of its codons. The correlation between the backwards-calculated and experimental mRNA levels was high (R_p_ = 0.65, *N* = 6367, *P* < 10^−^^6^) and almost comparable with those measured between data from different transcriptome data sources (0.74 < R_p_ < 0.85). This result indicates that the 64 codons carry with them the required transcriptional information to estimate the relative level of cellular mRNA with an acceptable accuracy.

**Figure 2.**
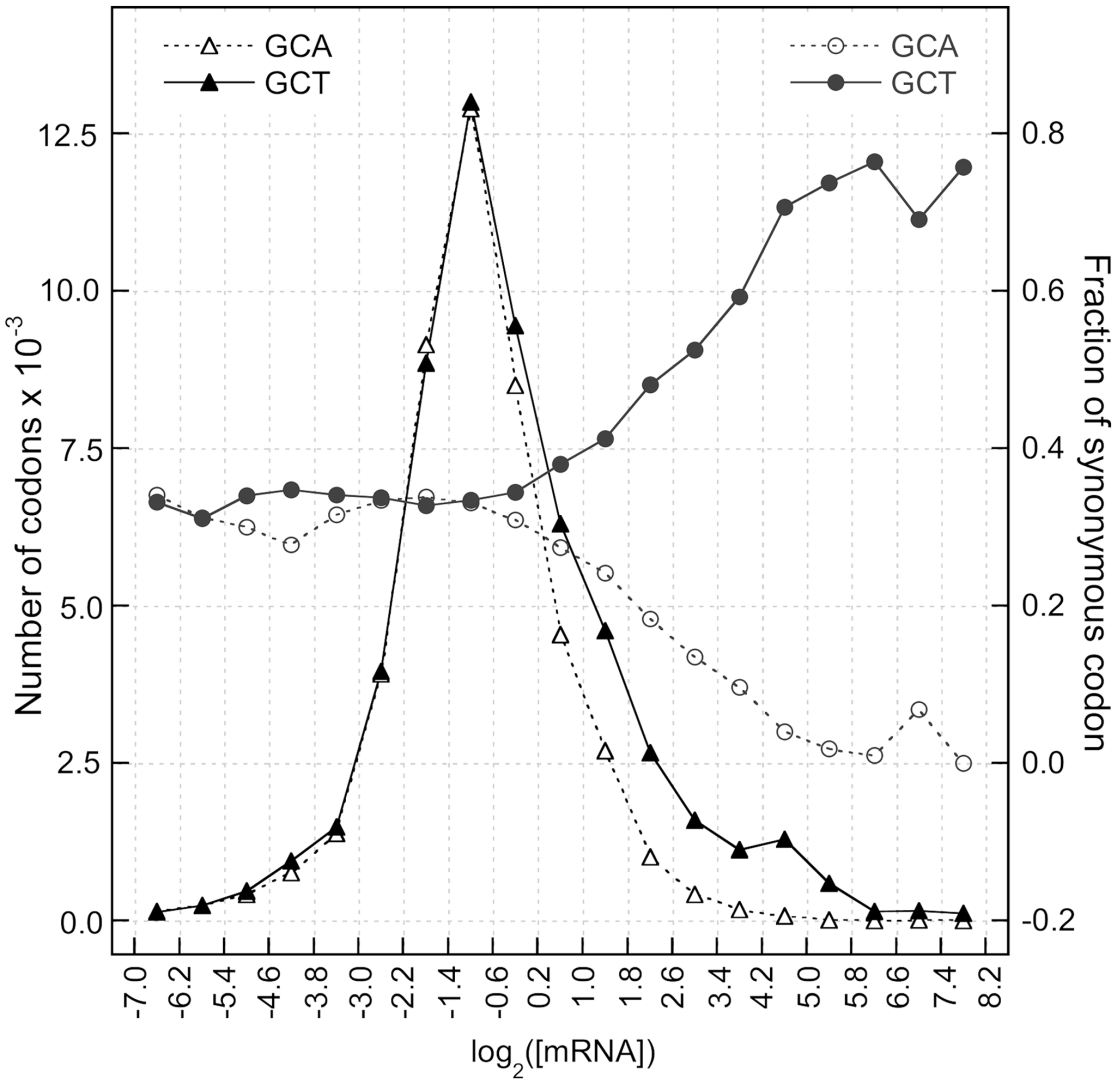
Frequency distribution and synonymous fraction of GCA and GCT. Frequency distribution (triangles) and synonymous fraction (circles) of GCA (open markers) and GCT (closed markers) with categories of transcript level. GCA and GCT are synonymous codons of the 4-fold degenerate amino acid alanine.

To exclude the possibility that the transcript levels associated with codons could be compatible with a neutral model in which mutational biases are the only active forces, we simulated 5000 yeast genomes with the codon probabilities at each synonymous site equal to their relative frequencies in the whole genome, but we left unchanged the transcript level assigned to each gene. The frequency distribution of the codon transcript level in simulated sequences showed that the levels of transcription in native codons is statistically incompatible with a neutral model (|Z-score| ranging from 7.6 to 77.5) (Supplementary Figure S1). We also verified that such a spread was not caused by outliers. This is illustrated in the graph in Figure 2 (triangles) that shows the frequency distribution of the transcription levels for the synonymous codons GCT and GCA. GCT is the preferred codon of the 4-fold degenerate alanine in the highly transcribed genes (major codon). Its distribution is asymmetric with respect to its synonymous minor codon (GCA). The asymmetry consists in an excess of the major codon (GCT) in the classes of genes with an average of >0.5 transcripts per cell. Because the two codons present an equal GC-content, which should minimize the possible biases due to mutational forces, this result suggests that selection on codon bias acts positively by favoring major codons in highly expressed genes rather than negatively by inhibiting the use of minor codons. The result is more apparent when the relative frequencies of synonymous codons are plotted as a function of the transcription level category (Figure 2, circles). The graph distinguishes three different classes of genes:

- lowly transcribed genes (mean number of mRNA molecules per cell up to 0.5) displaying an approximately equal level of codon bias,
- moderately transcribed genes (0.5 ≤ mRNA per cell ≤ 32) with the change of relative codon frequencies being almost linear with the transcript level and
- highly transcribed genes (mRNA per cell >32) characterized by strong codon bias.

The three categories of lowly, moderately and highly transcribed genes represent ∼49.3, 50.1 and 0.5% of the total number of codons, 44.8, 53.9 and 1.2% of the gene copy number and 4.9, 61.3 and 33.7% of the cellular transcripts, respectively. To better characterize the mutational and selective contributions to codon bias, we separately analyzed the lowly, moderately and highly transcribed genes.

### The codon bias of lowly transcribed genes is accurately predicted by the intergenic GC-content

**Figure 3.**
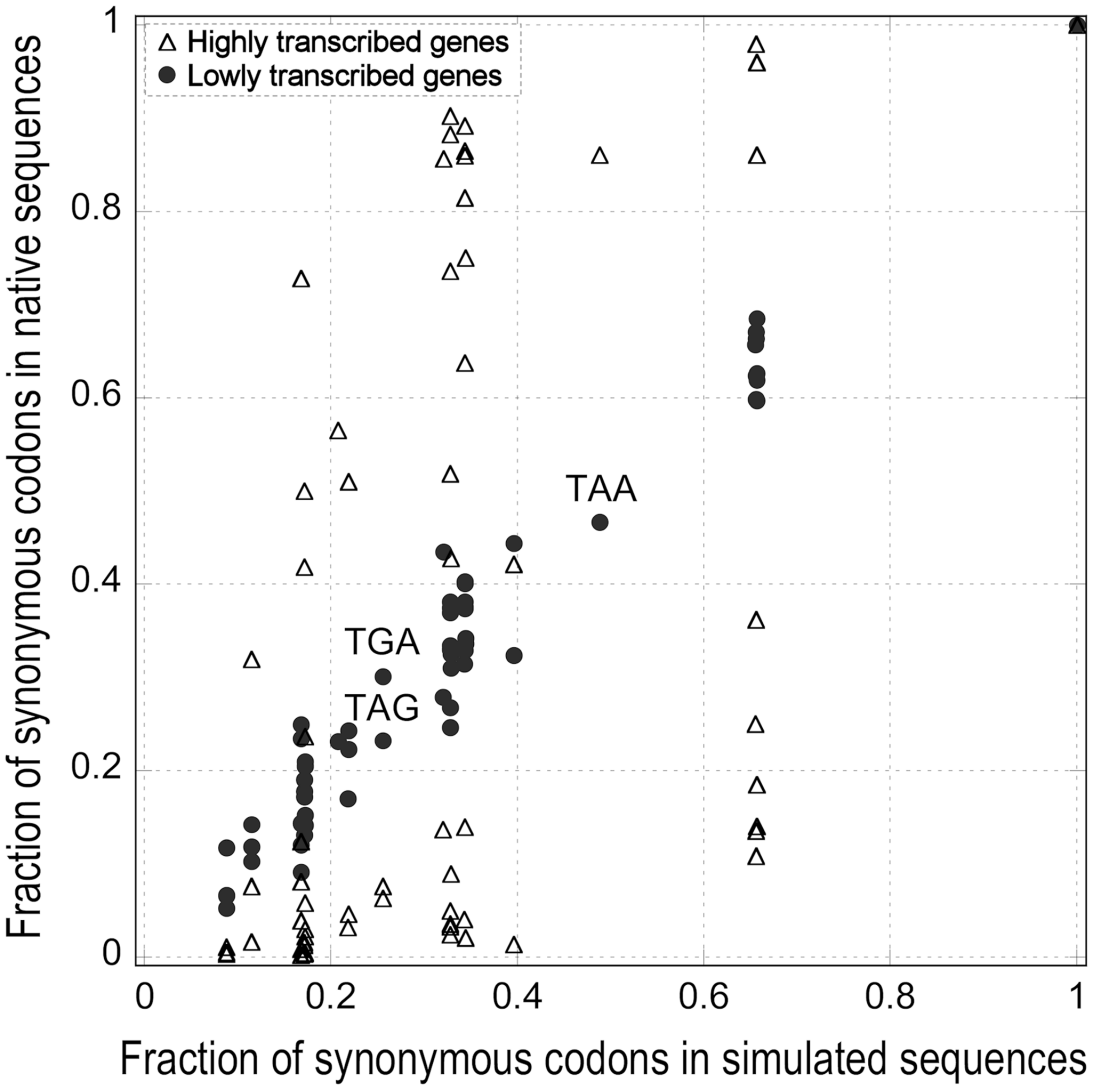
Fraction of synonymous codons in native and simulated sequences. Scatter plot of codon fractions in synonymous sets of lowly (closed circles) and highly transcribed (open triangles) genes versus codon fractions expected by coding sequences with GC-content equal to that of intergenic sequences.

We compared the relative frequency of the synonymous codons expected on the basis of the GC-content of intergenic regions with the relative codon frequencies of the lowly transcribed genes. As illustrated in Figure 3 (closed circles), the correlation analysis clearly shows that the codon bias of the lowly transcribed genes is accurately predicted by the GC-content of the intergenic regions (R_p_ = 0.98). In contrast, the codon bias of highly transcribed genes is scarcely correlated with that expected by the intergenic GC-content (Figure 3, open triangles).

### The codon bias of moderately transcribed genes correlates linearly with the mRNA levels

**Figure 4.**
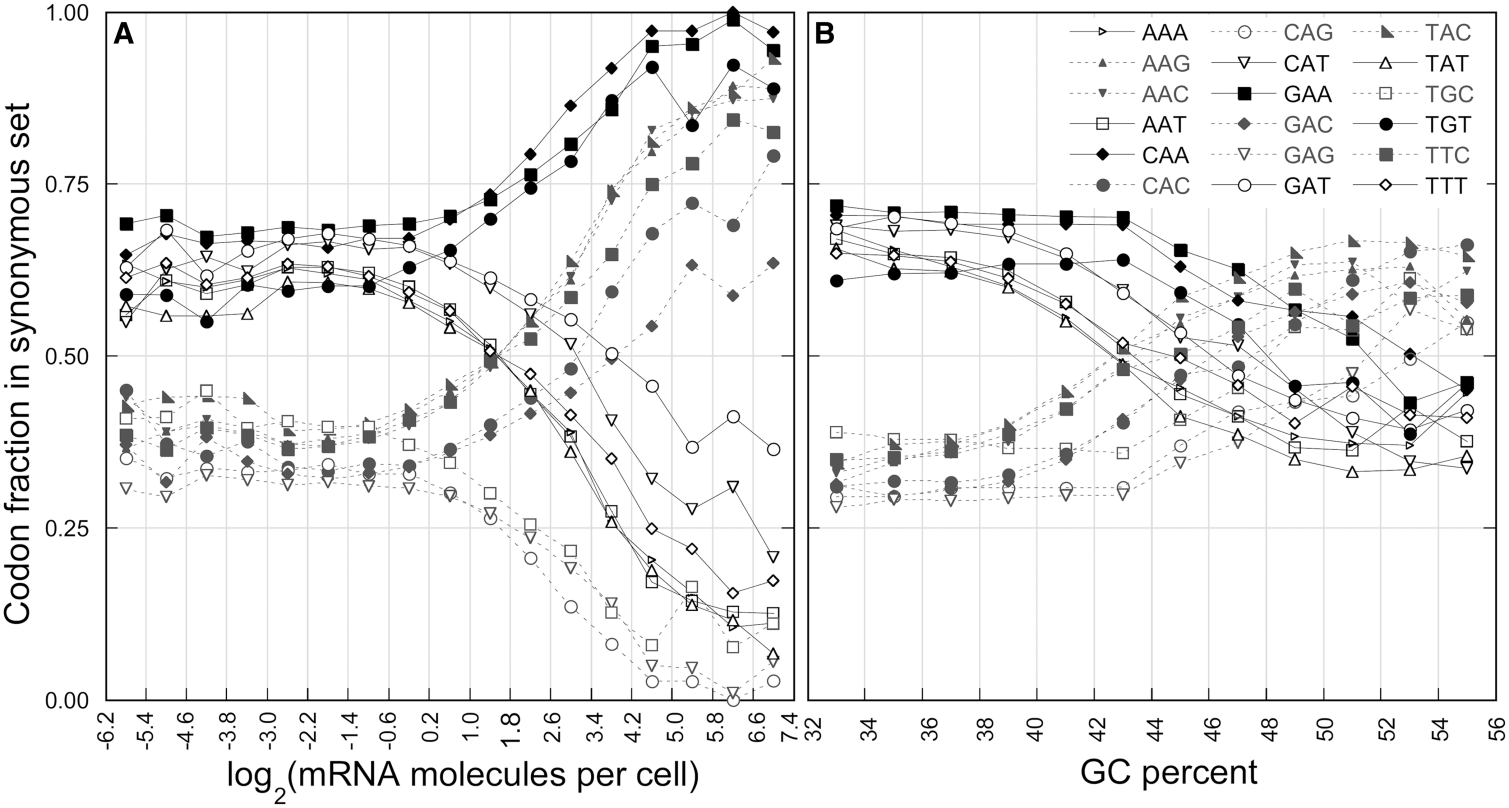
Change of synonymous fractions in 2-fold degenerate amino acids. Change of synonymous fractions of major (closed symbols) and minor (open symbols) codons with transcript level (panel **A**) and GC-content (panel **B**). The plot illustrates the nine 2-fold degenerated amino acids. The most and least frequent codons in lowly transcribed genes are in solid and broken lines, respectively.

The moderately transcribed genes present a bias for each set of synonymous codons that correlates almost linearly with the transcription level. Starting from the codon bias associated with the global GC-content, selective forces exert a pressure on codon choice that increases linearly with the gene expression level. Figure 4A shows the average relative use of synonymous codons of all 2-fold degenerate amino acids plotted against increasing transcription levels. The change of codon usage bias appears to be independent from the starting condition associated with mutational biases. In some cases, selection enforces mutation-induced codon bias (solid lines-closed symbols and broken lines-open symbols in Figure 4A), whereas in other cases, it forces codon frequencies in the opposite direction (solid lines-open symbols and broken lines-closed symbols in Figure 4A).

### The codon bias of the most transcribed genes is prevalently determined by natural selection, but it is also shaped by GC-content

In Figure 4A, we can observe that the preferred codons in the highly transcribed genes (major codons) reach a relative frequency of almost 100% only when they are also favored by mutational forces (solid lines-closed symbols in the figure). The other major codons reach a maximum relative frequency ranging from 60 to 90% (broken lines-closed symbols in the figure) with the lowest values associated with those codons with more than one G + C in their sequence: GAC and CAC. This result indicates that in highly transcribed genes, although codon usage is prevalently shaped by selective forces, the effects of mutational biases are not negligible, especially if the two forces are antagonistic.

Based on the balance of the relative codon frequencies of the 2-fold degenerated families, we tentatively estimated the contribution of the two major categories of neutral and selective forces to the global codon bias as 95 and 5%, respectively (see ‘Materials and Methods’ section). In general, the number of genes that experiences a significant selection on codon bias is remarkably lower than those that present a codon bias prevalently driven by mutational biases. This is the reason why mutational forces are the strongest determinant of the global CUF in yeast, as shown by the strong correlation between codon usage of the whole genome and that of lowly transcribed genes (R_p_ = 0.99) (Supplementary Figure S2).

### The relationship between codon usage and different coding sequence properties

The relative use of synonymous codons in a gene varies with the GC-content (Figure 4B) in a different way than with the transcript level (Figure 4A). For example, the relative frequency of CAG decreases with the transcript level but increases with the GC-content; in contrast, the relative frequency of CAC increases in both cases. These differences at the single codon level emphasize the distinct origin and consequences of the two forces and can be useful to distinguish between the two major categories of mechanistic causes of codon bias in yeast. Taking advantage of the high number of publicly available data for *S. cerevisiae*, we analyzed the relationship of a series of properties of coding sequences with the codon usage changes associated with the GC content and transcript level. We define two coding sequence properties to be positively (or negatively) coherent if the significant changes of all the relative codon frequencies with the magnitude of the two properties correlate positively (or negatively).

### Intrinsic nucleosome occupancy and the RNA/DNA and DNA/DNA duplex stability of coding regions are associated with GC-content

The GC-content explains ∼50% of the variation in nucleosome occupancy *in vitro* (32). Moreover, nucleosomes act as general repressors of transcription elongation (33). Here, we analyze genome-wide data of intrinsic nucleosome occupancy obtained from published sources (25). These data measured the occupancy of genomic regions that is only dependent on DNA sequence because it was achieved by *in vitro* assembling procedures using purified histones and yeast genomic DNA. We ignored data of *in vivo* systems because gene expression activity causes nucleosome depletion in the cell (25), and therefore, occupancy was not directly attributable to the coding sequence properties. Our results show that the intrinsic nucleosome occupancy of the coding regions is scarcely correlated with the mRNA level [Spearman’s rank correlation coefficient (R_s_) = 0.231 and coefficient of determination (R^2^) = 0.050, *N* = 5730, *P* < 10^−10^] and strongly correlated with the GC-content (R_s_ = 0.834 and R^2 ^= 0.619, *N* = 5745, *P* < 10^−^^10^). In agreement with this finding, the variations of the relative frequencies of synonymous codons with nucleosome occupancy are positively coherent with those associated with GC-content (Supplementary Figure S3).

It has also been suggested that the thermodynamic stability of DNA/DNA and mRNA/DNA duplexes affects mRNA transcription (24). Here, we analyze the genome-wide data for the calculated thermodynamic stability of DNA/DNA and RNA/DNA duplexes in yeast obtained from published sources (24). The duplex stabilities are strongly and positively correlated with the GC-content (DNA/DNA: R_s_ = 0.947 and R^2 ^= 0.900, *N* = 6543, *P* < 10^−^^10^; RNA/DNA: R_s_ = 0.791 and R^2 ^= 0.575, *N* = 6543, *P* < 10^−^^10^) and weakly correlated with the mRNA level (DNA/DNA: R_s_ = 0.220 and R^2 ^= 0.029, *N* = 6350, *P* < 10^−^^10^; RNA/DNA: R_s_ = 0.229 and R^2 ^= 0.037, *N* = 6350, *P* < 10^−^^10^). In agreement with these findings, the variations of the relative frequencies of synonymous codons with the duplex stabilities are positively coherent with those associated with the GC-content (Supplementary Figure S3).

### The folding structural properties, codon complementarity and three-base periodicity of coding sequences are associated with transcription level

**Figure 5.**
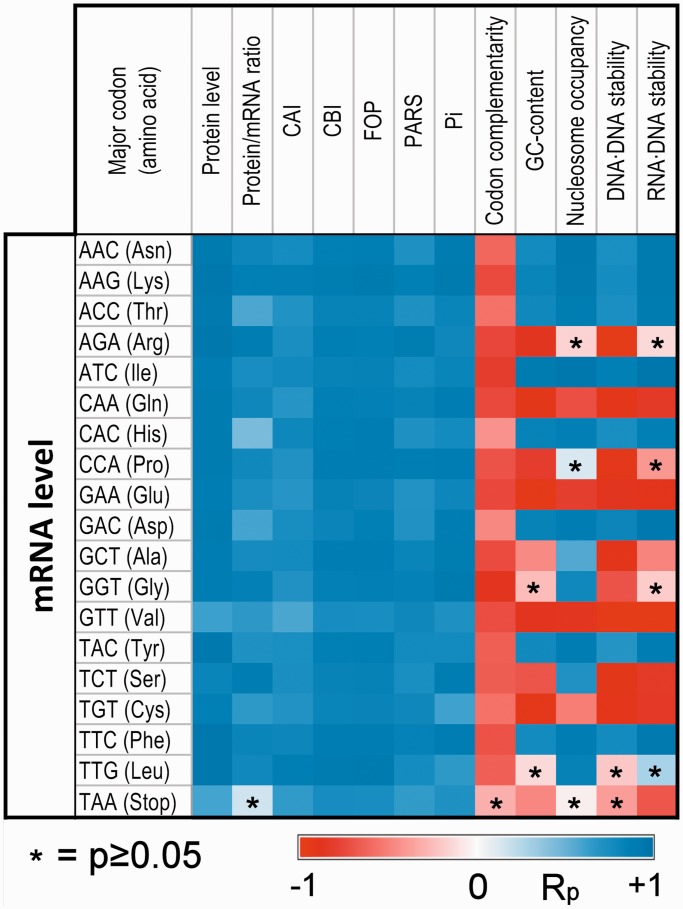
Comparison of the variation of the relative frequency of synonymous codons. Pearson’s correlation coefficients (R_p_) between the fraction change of major codons with increasing mRNA level and the fraction changes with increasing coding sequence properties: GC-content, CAI, Fop, CBI, cellular protein abundance, protein/mRNA ratio, codon complementarity, Pi (three-base periodicity index), PARS score, intrinsic nucleosome occupancy and thermodynamic stability of DNA/DNA and RNA/DNA duplexes (see ‘Materials and Methods’ section). For protein level and protein/mRNA ratio, we used a high-confidence data set of 408 genes (see ‘Materials and Methods’ section).

PARS is an experimental technique for the analysis of RNA secondary structure on a genome-wide scale (27). PARS measures the likelihood of a nucleotide in an mRNA molecule to be in a double-stranded conformation. Distinct from nucleosome occupancy, the average PARS score of an mRNA is strongly and positively correlated with the transcript level (16) (R_s_ = 0.642 and R^2 ^= 0.426, *N* = 2993, *P* < 10^−^^10^) and weakly with the GC-content (R_s_ = 0.311 and R^2 ^= 0.100, *N* = 3000; *P* < 10^−^^10^). Indeed, changes of codon fraction with increasing PARS score are positively coherent with those of mRNA levels (Figure 5 and Supplementary Figure S3).

Another property that is principally correlated with mRNA abundance is the level of codon complementarity. Here, we introduce this simple index that measures the fraction between the number of codon pairs in a gene that are Watson–Crick complementary and the total number of codons (see ‘Materials and Methods’ section). The complementarity score is negatively and weakly correlated with the GC-content (R_s_ = −0.208 and R^2 ^= 0.036, *N* = 6579, *P* < 10^−^^10^) and negatively correlated with the mRNA level (R_s_ = −0.307 and R^2 ^= 0.093, *N* = 6365, *P* < 10^−^^10^). Moreover, the variations of the relative frequencies of synonymous codons with codon complementarity score are negatively coherent with those associated with mRNA levels (Figure 5 and Supplementary Figure S3).

A third property related to the mRNA level is the Pi index, which measures the three-base periodicity of a coding sequence expected by its codon frequencies (26). The Pi score correlates more strongly with the mRNA level (R_s_ = 0.410 and R^2 ^= 0.188, *N* = 6365; *P* < 10^−^^10^) than with the GC-content (R_s_ = 0.206 and R^2 ^= 0.043, *N* = 6579; *P* < 10^−^^10^), and the change of codon usage with Pi score is coherent with that of the mRNA level (Figure 5 and Supplementary Figure S3).

The protein/mRNA ratio is also coherent with the mRNA level (Figure 5 and Supplementary Figure S3) and will be analyzed in detail later in the text.

Finally, all the codon bias indices calculated by the software CodonW (29): CAI, Fop, Nc and CBI were coherent with the mRNA level (Figure 5 and Supplementary Figure S3).

### The relationship between codon usage and the folding stability of coding sequences

PARS score is evaluated at nucleotide resolution (27), representing a measure of the likelihood of a nucleotide to be in a double-stranded conformation. In calculating the PARS score associated with each codon, we can proceed in two different ways. We can assign at each codon either the average PARS score of the mRNA where the codon is found or the average score of the three nucleotides that compose the codon. For simplicity, we termed the two codon indices as PARS-gene and PARS-nucleotide, respectively. We found that both indices demonstrate the existence of a strong relationship between mRNA structure and codon bias. The average PARS-gene score of each codon, calculated over all the 3000 analyzed transcripts, can be used to predict the major codons of all the synonymous families, including stop codons: the codons with the highest average PARS-gene score within its synonymous set are those preferred in the highly transcribed genes (Supplementary Table S1). Although the values of average PARS-gene scores associated to the 64 codons are well correlated with codon bias, their spread is very low when compared with that of the average PARS-nucleotide scores [standard deviation (s.d.) is 0.045 and 0.423, respectively]. This means that the distribution of codons within an mRNA molecule is strongly associated with the local folding stability. To quantify the spread of average PARS-nucleotide scores, we simulated 1000 sets of the 3000 CDSs used for PARS analysis by a random shuffle of the codons inside each CDS. The only difference between native and simulated sequences was the order but not the frequencies of codons. For each set of the 3000 simulated CDSs, we computed the s.d. of the average PARS-nucleotide scores associated with the 64 codons. The 1000 values of s.d. exhibited a distribution that was well approximated by a normal distribution with a mean of 0.047 and an s.d. of 0.0018, which was significantly incompatible with the s.d. of the native sequences (0.423). Therefore, the result shows a non-random distribution of codons among the regions of mRNA that have different folding structure stabilities. To test whether this non-random association between local structural stability and codon choice is correlated with the global structural stability of mRNAs and with the transcript levels, we computed the expected PARS value of each transcript from the average PARS-nucleotide values of each codon. The predicted PARSE score of transcripts was significantly correlated with its real PARSE score (R_p_ = 0.59, *N* = 3000, *P* < 10^−^^8^) and with the transcript levels (R_p_ = 0.31, *N* = 2993, *P* < 10^−8^).

Finally, to better clarify the origin of PARS-nucleotide scores, we calculated the average PARS score of the four nucleotides from the 3000 analyzed CDSs: A = 0.034, C = 0.844, G = 0.026 and T = 0.540. Then, we estimated the PARS score of each codon from the average score of its three nucleotides [e.g. GAC equal to (0.026 + 0.034 + 0.084)/3]. The estimated PARS scores and the real average PARS-nucleotide scores of codons are strongly correlated (Supplementary Figure S4) (R_s_ = 0.78 and R^2 ^= 0.64, *N* = 64, *P* < 10^−^^8^), showing that ∼64% of the relative average PARS-nucleotide score of each codon can be predicted by its nucleotide composition.

### The relationship of CDS length with PARS score and transcript level

We analysed the relationship of CDS length with PARS score and transcript level. We found that CDS length is weakly but significantly correlated with transcript level (R_s_ = −0.119, R^2 ^= 0.011, *N* = 6365, *P* < 0.00001) but does not appear correlated with PARS score (R_s_ = 0.091, R^2 ^= 0.0004, *N* = 3000, *P* = 0.28). Moreover, as shown in Supplementary Figure S5, the variations of the relative frequencies of synonymous codons with mRNA length are much smaller than those with the other CDS properties analyzed in this work.

### The thermodynamic folding stability of introns is correlated with mRNA level and codon bias

Introns are transcribed but not translated because, before protein synthesis, they are removed from pre-mRNA transcripts by the spliceosome, a multimegadalton ribonucleoprotein complex. The spliceosome can be assembled during transcription elongation (34) and, in yeast, most splicing events take place cotranscriptionaly near gene ends (35). Therefore, if mRNA structure plays a role in the optimization of transcriptional processes, the correlation between the folding stability and the transcript level should also apply to intronic regions.

**Figure 6.**
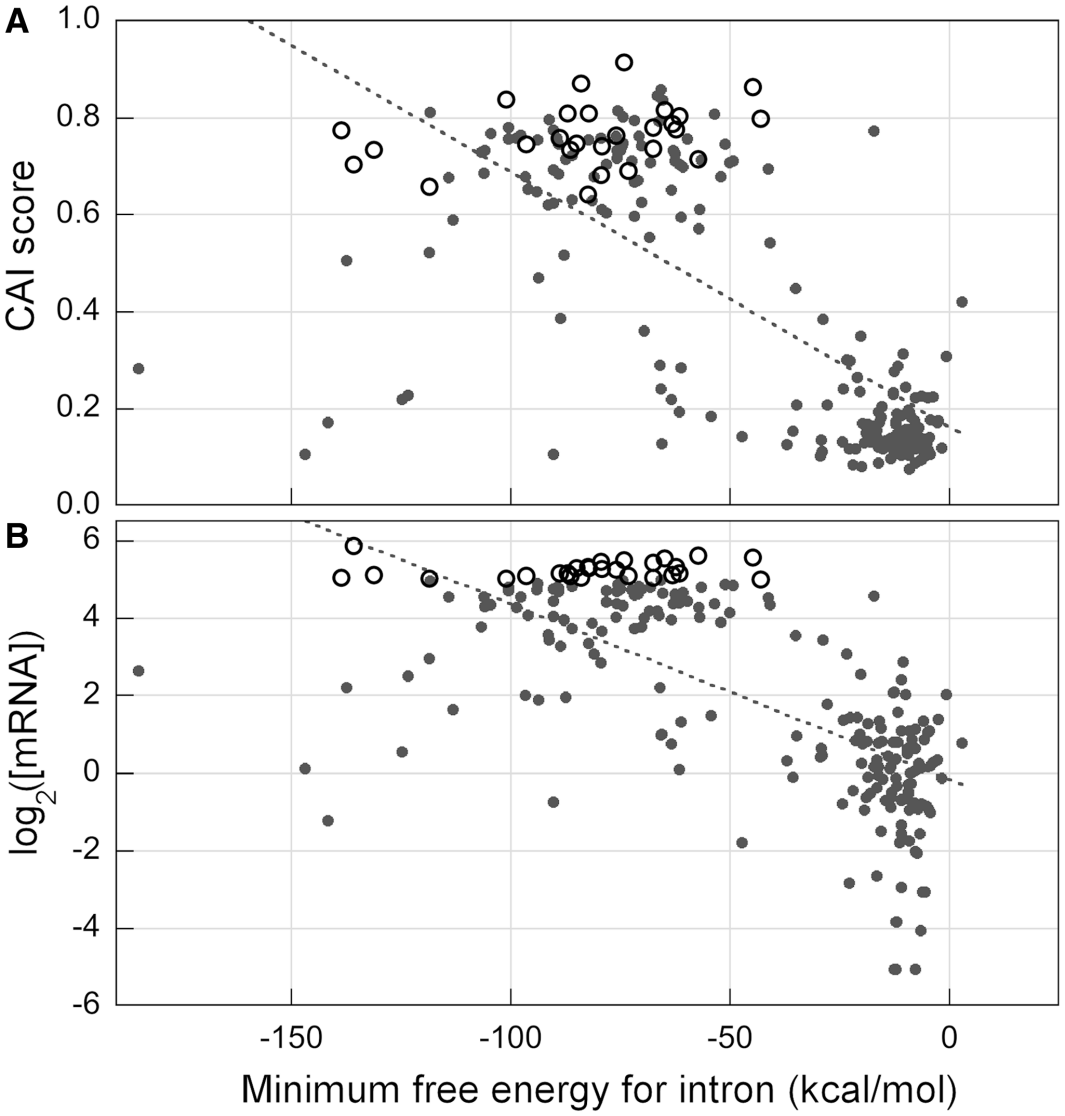
Correlation of CAI score and mRNA level with the minimum free energy for intron sequences. Scatter plots showing the correlation of CAI score (panel **A**) and mRNA level (panel **B**) with the free energy of intron structures predicted using Quickfold from Mfold web server. Highly transcribed genes (mRNA per cell >32) are indicated by open circles. The broken line represents the linear regression line. The transcript concentration is expressed in log_2_-transformed molecule per cell.

To check whether the folding structural stability of introns is correlated with the PARS score of coding sequences, with transcript levels and with codon bias, we computed the minimum free energy (MFE) structure of intronic sequences using the Quikfold option of the DINAMelt web server (*RNA* 3.0) (28). The result shows that the minimum free energy (ΔG) for each intron is strongly and negatively correlated with the mRNA level (R_s_ = −0.693 and R^2 ^= 0.461, *N* = 235, *P* < 10^−^^5^) (Figure 6B), and significantly correlated with the PARS score of coding regions (R_s_ = −0.362 and R^2 ^= 0.09, *N* = 205; *P* < 10^−^^5^). Surprisingly, we also found that the minimum energy folding for each intron is strongly correlated with codon bias scores of coding sequences (Figure 6A): CAI (R_s_ = −0.687 and R^2 ^= 0.515, *N* = 235, *P* < 10^−^^5^), CBI (R_s_ = −0.688 and R^2 ^= 0.531, *N* = 235, *P* < 10^−^^5^), Fop (R_s_ = −0.686 and R^2 ^= 0.515, *N* = 235, *P* < 10^−^^5^), CBI (R_s_ = −0.687 and R^2 ^= 0.526, *N* = 235, *P* < 10^−^^5^) and Nc (R_s_ = 0.670 and R^2 ^= 0.479, *N* = 235, *P* < 10^−^^5^). The MFE of a RNA sequence is a function of its length, nucleotide composition and nucleotide order. Thus, when intron is bound to the assembled spliceosome and, therefore, its short-range interactions with adjacent exon sequences are constrained by the ribonucleoprotein complex, its folding stability should increase with the length of its sequence. It has been previously reported that intron length in yeast genes is strongly and positively correlated with codon bias and the level of gene expression (36). Here, we found a strong correlation between the MFE and the length of intronic sequences (R_s_ = −0.959 and R^2 ^= 0.479, *N* = 249, *P* < 10^−^^5^) showing that almost all the computed MFE is attributable to sequence size. We also found that the length as well as the MFE of intronic sequences is weakly correlated with CDS length: R_s_ = −0.192 and R^2 ^= 0.03, *N* = 249, *P* = 0.0066 and R_s_ = 0.202 and R^2 ^= 0.03, *N* = 249, *P* = 0.0059, respectively. Therefore, although the sequence length of introns and exons are very weakly and negatively correlated, their folding stabilities are strongly and positively correlated. The correlation of transcription level with the length-dependent MFE of introns and the PARS score of exons may involve long-range effects of pre-mRNA folding structures or structure-dependent interactions with the components of the transcription elongation complex. These results suggest that the association between the mRNA folding stability, codon bias and the transcript level occurs, at least partially, before the splicing of pre-mRNA.

### The relationship between codon complementarity and codon usage bias

**Figure 7.**
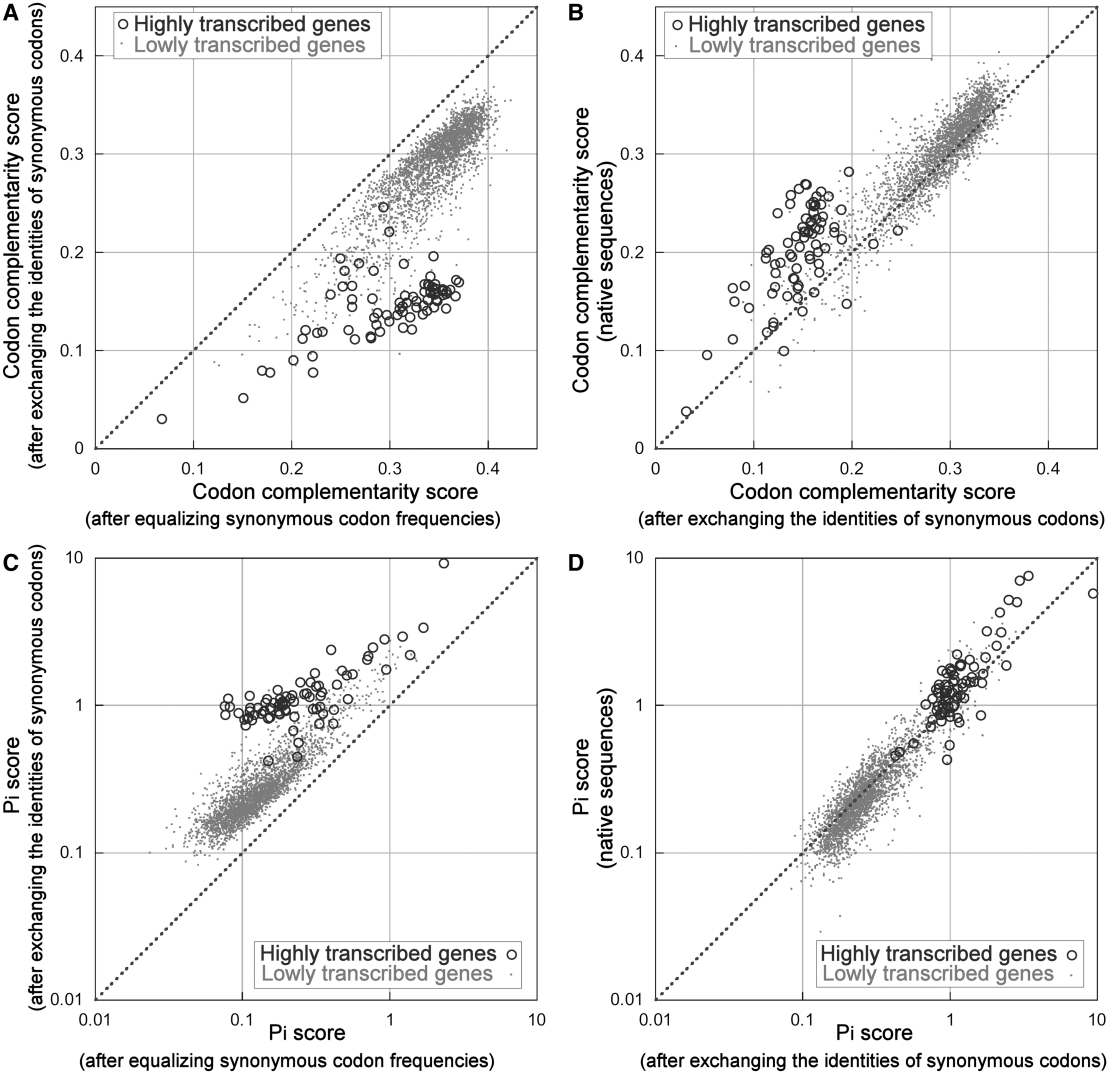
Comparison of codon complementarity and Pi scores in simulated and native sequences. Two sets of genomic coding sequences were simulated from native mRNA: in the first set, the frequencies of synonymous codons within each gene were equalized; in the second set, the identities of synonymous codons were exchanged randomly and uniformly in all genes. The scatter plots report the comparison of codon complementarity (panel **A**) and Pi (panel **C**) scores of the gene in the two simulated sets of sequences. The scatter plots of panel **B** (codon complementarity) and **D** (Pi score) show the comparison between native and codon exchanged sequences. Circle and point markers indicate highly and lowly transcribed genes, respectively. The broken line corresponds to equal values on both axes.

As reported earlier in the text, the codon complementarity score of coding sequences is negatively correlated with the transcript level and with the codon bias associated with selective forces. To investigate the relationship between codon complementarity and codon bias, we simulated two different sets of CDSs from native mRNAs. In the first set, we simulated a genome without codon bias by equalizing the frequencies of synonymous codons within each native CDS. In the second set, we preserved the codon bias of each synonymous set but randomly exchanged the identities of synonymous codons uniformly in all coding sequences. For example, in a simulation, we substituted all codons of glycines in the following way: all GGA were changed to GGG, all GGC to GGT, all GGG to GGC and all GGT to GGA. The differences between the two simulations are attributable to the codon bias but not to the identity of minor and major codons because, in the sequences with exchanged codons, the score is averaged over 20 different simulations. Figure 7A reports the comparison of the complementarity scores calculated for the CDSs of the two simulations. From the graph in Figure 7A, the complementarity scores of the two simulated sets of CDSs are strongly and positively correlated. This is more obvious if we consider only the lowly transcribed genes (points in Figure 7A). This shows that a consistent part of the complementarity is not due to codon bias but is generated by the combination between the structure of the genetic code and the amino acid composition of a protein. From the plot of Figure 7A, it is also observable that, in all CDSs, codon bias determines a decrease of codon complementarity that, in agreement with the above correlational studies, is higher in highly than in lowly transcribed CDSs. Figure 7B compares the complementarity of native CDSs with the average complementarity of simulated sequences with codon identity exchanged. In this case, the differences are only attributable to the specific identities of major and minor codons used in the native sequences and not to the codon bias that is preserved in the simulated sequences. The result shows that the choice of minor and major codons in native sequences produces a higher increase of complementarity in highly than in lowly transcribed CDSs. In summary, although an increase of codon bias generally produces a reduction in codon complementarity of CDSs, the choice of major codons opposes this reduction in highly expressed CDSs. The result suggests that the need to maintain an optimal number of complementary codons in the most expressed genes may affect the choice of major codons.

### The relationship between three-base periodicity and codon usage bias

As reported earlier in the text, the Pi score of yeast CDSs is positively correlated with the transcript level and with the codon bias associated with selective forces. To investigate the nature of the relationship between the Pi score and codon bias, we simulated two different sets of CDSs from native mRNAs using the same procedures reported previously for the analysis of codon complementarity: one set with synonymous codon frequencies equalized and a second set with synonymous codon identities randomly and uniformly exchanged. The results are illustrated in Figure 7 (panels C and D). As discussed earlier in the text, for codon complementarity, the correlation between the two simulated set of sequences, illustrated in Figure 7C, indicates that an important contribute to the Pi score of a CDS is intrinsically due to the structure of genetic code and the amino acid composition. The codon bias associated with mutational biases of lowly transcribed genes causes an almost constant increase in Pi. Consistent with the aforementined correlation analysis, the increased codon bias of highly transcribed genes, which is associated with selective forces, is reflected in an increased Pi score. Moreover, a weak but significant (*P* < 10^−^^5^) increase of periodicity in the native sequences is also determined by the choice of major codons in highly transcribed genes (Figure 7D). Therefore, the choice of major codons reinforces the effect determined by the magnitude of codon bias on the three-base periodicity of coding sequences.

### The number of complementary major codons is significantly high

The major codon was defined as the most frequent synonymous codon of an amino acid in highly transcribed genes. We found that 10 major codons, of the 18 degenerate sense families, are complementary. The number of complementary major codons is significantly high (*P* < 0.05), as estimated by 100 000 simulations in which the major codon of each amino acid was chosen at random.

### Stop and sense codons are under similar selection pressure

The three stop codons TGA, TAA and TAG are the signals of translation termination. Distinct from the sense codons, they are normally not decoded by an aminoacyl-tRNA but are directly recognized by protein release factors (37) that facilitate the termination of translation and the release of finished protein from ribosome. If a pre-translational force is the primary cause of selection on codon bias, we should observe an almost equal pressure over stop and sense codon families. In contrast, if the selection on codon bias is principally associated with the tRNA frequencies, the stop codons should not be under the same selective pressure of sense codons. Here, we compare the relative use of stop and sense codons at increasing transcript levels.

**Figure 8.**
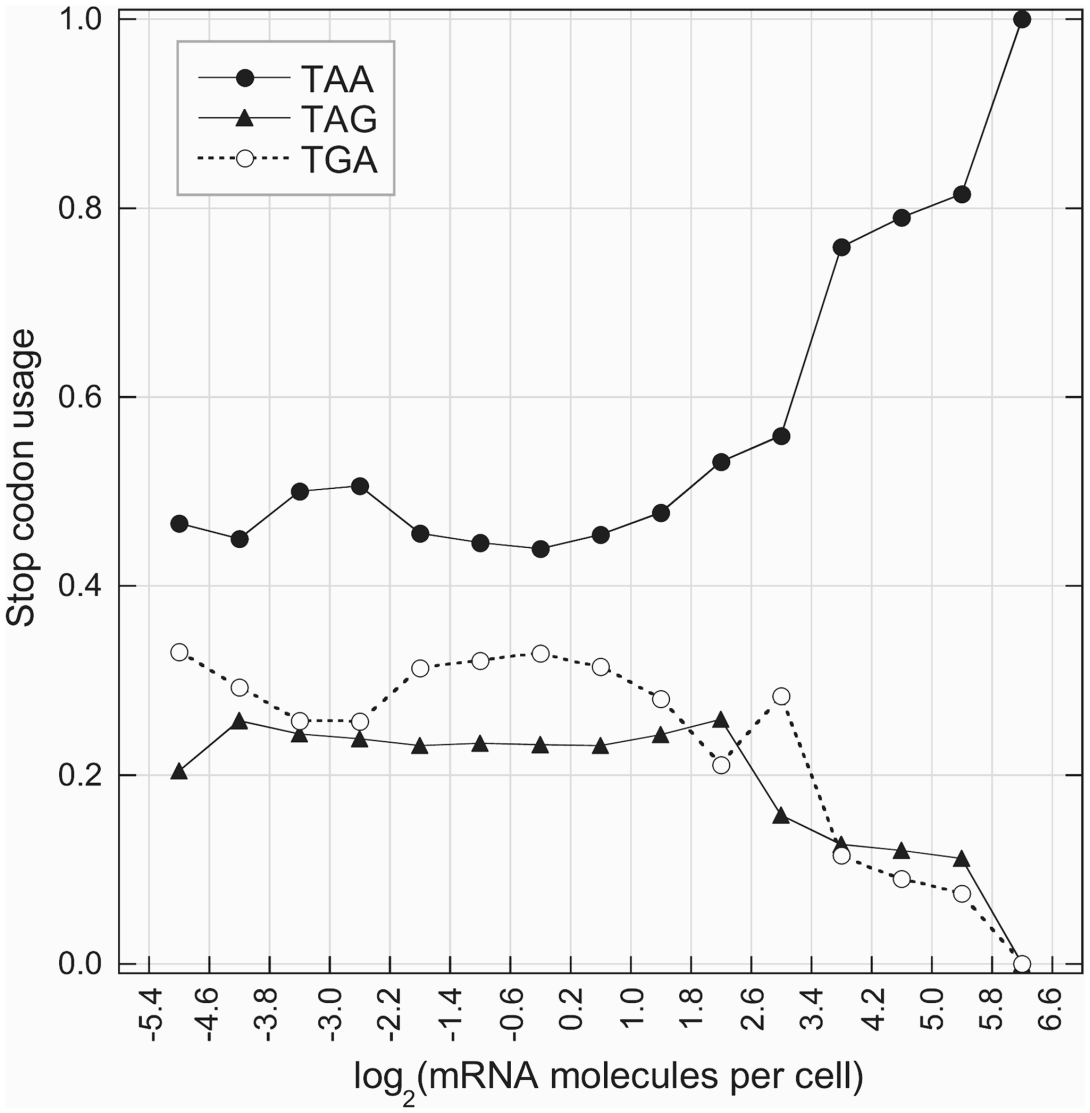
Change of translational stop signal as a function of mRNA level. For these graphs, we used high-confidence data set of 408 genes (see ‘Materials and Methods’ section).

At the genome level, stop codons are unequally frequent: TAA, TAG and TGA are present at 47, 23 and 30%, respectively. According to the sense codons, the relative frequencies of stop codons in lowly transcribed genes are strongly compatible with those expected for GC-content of intergenic sequences (see Figure 3). Moreover, as illustrated in Figure 8, at increasing transcription levels, the variation of the relative frequencies of the three stop codons is strongly consistent with that of sense codons. In the highly transcribed genes, the ‘major’ codon of stop signal (TAA) reaches almost 100% of the relative frequency. In contrast with the hypothetical major role of tRNA abundance in the origin of codon bias, this result strongly suggests that sense and stop codons are under similar selective pressure.

### Protein/mRNA ratio and codon usage

**Figure 9.**
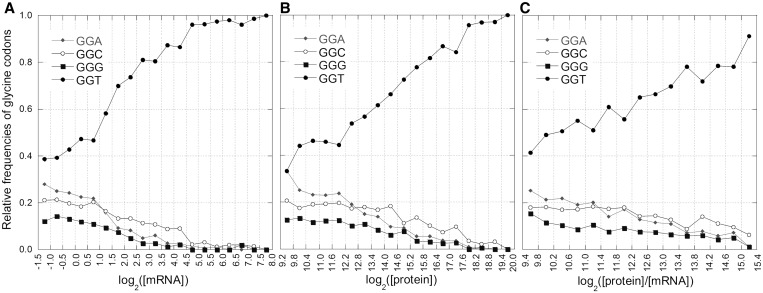
Codon usage changes of the 4-fold degenerate glycine. Codon usage changes of the 4-fold degenerate glycine as a function of mRNA level (**A**), protein level (**B**) and protein/mRNA ratio (**C**). Concentrations are in units of molecules per cell. For these graphs, we used high-confidence data set of 408 genes (see ‘Materials and Methods’ section).

We used a high-confidence data set of protein and mRNA cellular levels of 408 genes (26) to test the hypothesis that the number of protein copies per mRNA molecule is constant across different transcript levels. The results indicated that the protein/mRNA ratio significantly rises with the transcript level as described in the Results section of Supplementary data. Moreover, the variation of the relative frequencies of synonymous codons with the level of protein per mRNA is coherent with that observed with mRNA (Figure 5 and Supplementary Figure S3). As an example, Figure 9 illustrates the codon usage changes of the amino acid glycine with increasing levels of mRNA, protein and protein/mRNA. Interestingly, in contrast with sense codons, the variation of the codon usage of the stop signal with protein/mRNA ratio is not correlated to the variation with the mRNA level (R_p_ = 0.16, *P* = 0.48, Figure 5). This is consistent with a role of tRNA pool in favoring the relationship between protein yield and transcript abundance.

### Codon bias within a gene

We analyzed the weight of the contributions of different intragene regions to the whole codon bias. The results indicated that the major causes of codon bias cannot be found in mechanisms that act differently along the mRNA molecule (Supplementary Data and Supplementary Figure S6).

### Codon usage and tRNA levels

In yeast, as in some other organisms, there is a strong positive correlation between the codon usage of highly expressed genes and tRNA abundance (14,38). Because complete direct data of tRNA abundance in yeast are not available, as an estimate, we used the relative number of tRNA genes, which has been shown to be an acceptable approximation (38,39). As widely reported in the literature, we found a positive correlation between codon usage and the tRNA number of genes. However, five codons that strongly increase their frequencies with mRNA level lack the cognate tRNA with the perfect Watson–Crick complementary anticodon: TGT (cysteine), GGT (glycine), ATC (isoleucine), ACC (threonine) and GTC (valine). The frequency increase of these codons with transcription level does not appear to be consistent with the hypothesis that major codons are selected for matching the most abundant tRNA. For example, as shown in Figure 9A, although the anticodons for the 4-fold degenerate glycine are CCC, GCC and TCC, at a progressive increase of transcription level, the relative use of their perfectly paired codons GGG, GGC and GGA drops to ∼0%. In contrast, the frequency of the major codon GGT, which is the only glycine codon lacking a perfectly paired anticodon, increases to ∼100%. The case of the 2-fold degenerate amino acids cysteine, aspartic acid and histidine is particularly interesting. All three amino acids present only one anticodon, which begins with G and is constituted by two G + C nucleotides: GCA, GTC and GTG, respectively. However, as shown in Figure 4A, with increasing expression levels, a wobble pairing (U:G) is favored over the Watson–Crick pairing (C:G) at the third codon position of cysteine sites, whereas the C-ending codons are preferred for aspartic acid and histidine. If codon usage is adapted to tRNA profile, these incongruent selections at the third codon position indicate that the choice between G:U and G:C should be under weak selection, in agreement with the wobble rules (40). In this case, we should consider this weak selective force stronger than any other possible mechanism influencing codon usage, such as mutational biases and mRNA structural constraints. Conversely, wobble rules explain the frequencies of iso-accepting tRNA in the model that considers the tRNA population as being adapted to match codon usage of highly expressed genes. All nine tRNA anticodons that show Watson–Crick complementary base pairing to a major codon are the most frequent of their respective amino acid sets. When perfectly paired anticodons are lacking, the iso-accepting anticodons with wobble pairing to the major codons are the most abundant. This asymmetric coevolution between tRNA pool and codon bias appears more compatible with a model in which the relative abundances of tRNAs reflect their adaptation to codon usage rather than vice versa.

## DISCUSSION

In this study, an *in silico* analysis of codon usage was performed to test for a possible role of transcriptional mechanisms in shaping codon bias. The study was performed using *S. cerevisiae*, taking advantage of the high number of genome-wide data sources that are available for this organism and its favorable eukaryotic and unicellular nature. As a eukaryotic organism, yeast presents the simplicity of having the transcription and translation processes separated in time and space, in contrast with prokaryotes, in which translation occurs during transcription elongation with confusing physical reciprocal interference. Moreover, as a unicellular organism, selection on codon bias is not affected by the different expression profiles of differentiated cells.

The mutation-selection-drift balance model (5–7) is the general theory commonly invoked to explain codon bias. The theory assumes that the unequal frequencies of synonymous codons is generated by the balance between forces of mutational and selective mechanisms acting in a finite population: mutational biases uniformly affect the GC-content of the whole genome, selective forces primarily act on coding sequences with a strength that is gene specific. The principal cause of selection on codon bias is generally attributed to translational forces: codons with more abundant cognate tRNA are translated more efficiently or accurately because they reduce ribosome pausing during elongation and decrease the probability of incorporating incorrect amino acids (12,13,41).

In contrast with the consensus view, the results presented here emphasize the role of transcriptional against translational mechanisms in shaping the codon usage of highly transcribed genes.

First, we evaluated the relative contribution of natural selection and mutational biases to the origin of codon bias in yeast. We estimated that almost all (≈95%) of the codon bias of the whole genome may be attributable to mutational biases, although natural selection constitutes the dominant force that drives codon usage in highly expressed genes. We found that the codon bias due to mutational mechanisms appears to be mainly associated with the properties of the coding sequence that depend essentially on its primary structure, including intrinsic nucleosome occupancy or RNA/DNA and DNA/DNA duplex stability. In contrast, the sequence properties that are somehow connected with mRNA secondary structure, including folding structural stability (PARS score), codon complementarity and three-base periodicity (Pi score), appear to be correlated with codon bias associated with natural selection. The strong positive correlation between the PARS score of a transcript and its expression level has been reported and deeply discussed recently (16). The authors also found a strong correlation between the PARS score and the ribosome density, concluding that, at least partially, folding structure is connected to the efficiency of translation elongation. In our study, we found a clear relationship between selection on codon bias and PARS score. Our results show that, from PARS data, we can predict the major codons of all synonymous sets. Moreover, we found that the codon distribution within an mRNA molecule is non-random with respect to the local folding stability. This result agrees with an *in silico* analysis of human and mouse mRNA folding, in which the authors suggest that selection operates on synonymous codon sites for a more ordered and more stable mRNA secondary structure (42). The relationship between folding structure and codon bias is also consistent with the results of our correlational studies on three-base periodicity and codon complementarities. A three-base periodic pattern of mRNA secondary structure associated with codon usage has been reported for yeast (27) and mammals (42). It has been suggested that degeneracy of genetic code permits the regulation of the stability and periodicity of mRNA secondary structure and that periodicity in mRNA secondary structure favors the formation of intramolecular helices and a more compact transcript folding (42). Here, we found that both codon bias and the choice of major codons contribute to the higher three-base periodicity of highly expressed genes compared with lowly expressed genes. With regard to codon complementarity, it has been recently reported that the diminished fitness conferred by a synonymous mutation can be recovered by introducing a second synonymous substitution that restore base pairing in an mRNA stem (43). Also, in *Strongylocentrotus purpuratus*, the third positions of preferred codons paired signiﬁcantly more often in stems than in loops of mRNA secondary structure predictions (44). Here, we found that, in contrast to three-base periodicity, the codon complementarity decreases with increasing codon bias, but the choice of major and minor codons significantly contrast the effect of codon bias in highly expressed genes. These results suggest that the strength of codon bias and the choice of major codons may reflect different forces shaping codon usage. The increase of codon bias may be advantageous because it increases the periodicity and the structural stability of mRNAs and decreases codon complementarity, preventing the interference of not optimized intra-strand pairings during transcription and translation. In highly expressed genes, the reduction of codon complementarity by codon bias may become highly incompatible with the formation of stable folding structure, and for this reason, it could be compensated by the choice of suitable major and minor codons. We also found that the theoretically most stable folding structure of each intron exhibits a ΔG that is significantly and negatively correlated with the experimental folding structure stability of coding sequences, the transcript level and the codon bias. This suggests that the relationship between mRNA structure, codon bias and gene expression may take place, at least in part, before pre-mRNA splicing. Therefore, in addition to the inferred role of translation elongation (16), transcription optimization should also be considered as the cause of the relationship between mRNA structure and expression level. This conclusion agrees with the recent finding that nascent RNA structure reduces pausing and favors transcription elongation (17). As a whole, the clear relationship among transcript abundance, codon bias and the structural properties of introns and exons, together with the finding that mRNA structure influences transcription elongation (17), strongly suggests a role for transcription in shaping codon bias through structural constraints.

From the earliest studies approximately three decades ago, in which the authors suggested that codon usage bias might be the result of an adaptation to the tRNA pool (12,45), the fundamental question of whether codons are adapted to anticodons or vice versa is, to our knowledge, unresolved as yet. It cannot be excluded that, inversely, tRNA abundance reflects its adaptation to codon bias or that they have coevolved. This is a fundamental question regarding the role of transcriptional and translational mechanisms in the origin of codon bias.

Consistent with a contribution of transcription in selection on codon bias, we report some evidence supporting the idea that the tRNA pool is adapted to codon bias rather than vice versa. First, we found that the 3-fold degenerate set of stop codons, which normally are not decoded by an aminoacyl-tRNA but by a protein complex, are under a selection pressure strongly consistent with that of sense codons. Second, we found that codon bias is correlated with minimum folding energy of introns. Third, we observed that the unbalanced codon–anticodon coevolution is better explained by wobble rules if the most abundant tRNAs are selected by matching with major codons rather than vice versa. Fourth, all major codons begin to increase their relative frequencies at almost the same transcription level. This common response is difficult to explain whether the adaptation of each codon is affected by the base-pairing thermodynamics with its own cognate anticodons. A structural equilibrium involving extended regions of mRNAs appears to be more consistent with the homogeneous changes of codon frequencies with transcription levels. Finally, we found that major codons are significantly complementary to each other and that codon choice in highly expressed genes produces a significant increase of codon complementarity. There is no apparent explanation for such complementarity based on an adaptation of codon usage to the tRNA pool; however, it may reflect the requirement of an increased folding structural stability. In agreement with the hypothesis that the tRNA pool is adapted to codon usage rather than vice versa, the expression levels of individual tRNA species in humans are tissue-specific (46,47), providing evidence of the adaptability of tRNA pool to the different requests of transcript profiles. The coevolution of the tRNA pool and the codon usage of highly transcribed genes should favor the increase of translation efficiency in most expressed genes. We found a statistically significant increase of protein produced per mRNA at increasing levels of transcript. We also found that the changes of the relative frequencies of sense codons with the protein/mRNA ratio are coherently correlated with those of increasing mRNA levels. This association is not observed for the relative frequencies of stop codons, emphasizing the role of tRNAs in the relationship between the protein yield of a transcript and its codon usage. The participation of both transcriptional and translational forces in modulating the expression level by tuning codon usage is also consistent with some of the literature. In yeast, the level of highly expressed PGK protein and the steady-state mRNA levels decreased 10- and 3-fold, respectively, when major codons were replaced by minor codons (48). Codon optimization increases protein and steady-state mRNA levels in heterologous gene expression of *Aspergillus oryzae* (49) and *Aspergillus niger* (50). Substitutions of synonymous codons significantly increase papillomavirus L1 mRNA in transiently gene-transfected keratinocytes (51). Therefore, the increase in translational yield of the most abundant transcripts may be the force that favors selection of the most abundant tRNA. Additionally, the most abundant tRNAs may favor the uniform choice of major codons in all moderately and highly expressed genes.

## CONCLUSIONS

In conclusion, the results reported here suggest that transcriptional mechanisms may play a significant role in shaping the codon bias in yeast. mRNA secondary structure may be the link that connects the transcriptional activity with the codon bias in highly expressed genes. In a hypothetical model, natural selection favors those folding structures that are advantageous to the transcriptional and translational mechanisms of the most highly expressed mRNA by tuning codon bias and constraining the choice of major codons. The increase of codon bias is inherently associated with an increase of three-base periodicity that may favor the formation of intramolecular helices and a more compact transcript folding (42) and with a decrease of codon complementarity that may prevent the interference of non-optimized intra-strand pairings. The choice of major codons may be regulated by the most highly expressed genes through a mechanism that may include a contrasting reaction to an excessive reduction of codon complementarity. The adaptation of tRNA frequencies to the codon usage of the most expressed genes optimizes translation efficiency and drives the choice of the same major codons in all genes that undergo to selective pressure. In this model, the process that causes selection on codon bias starts with structural constraints of the most expressed genes, whereas the relative abundance of tRNAs favors the uniform use of the same major codons in different genes.

## SUPPLEMENTARY DATA

Supplementary Data are available at NAR Online, including [52–55].

## FUNDING

Consiglio Nazionale delle Ricerche (CNR). Funding for open access charge: CNR.

*Conflict of interest statement*. None declared.

## Supplementary Material

Supplementary Data
