# Supplementary material for: Selection on codon bias in yeast: a transcriptional hypothesis
Source: Nucleic Acids Res. 2013 Aug 13;41(20):9382–95. doi: 10.1093/nar/gkt740 (PMC3814355; doi:10.1093/nar/gkt740)
Supplement: Supplementary Data [file supp_41_20_9382__index.html]

Selection on codon bias in yeast: a transcriptional hypothesis — Selection on codon bias in yeast: a transcriptional hypothesis — Supplementary Data 

# Selection on codon bias in yeast: a transcriptional hypothesis

## Supplementary Data

files

**Files in this Data Supplement:**

- Supplementary Data - pdf file
