# Supplementary material for: Selection on codon bias in yeast: a transcriptional hypothesis
Source: Nucleic Acids Res. 2013 Aug 13;41(20):9382–95. doi: 10.1093/nar/gkt740 (PMC3814355; doi:10.1093/nar/gkt740)
Supplement: Supplementary Data [file supp_gkt740_nar-01679-v-2013-File011.pdf]

Supplementary data "Selection on codon bias in yeast: a transcriptional hypothesis"

RESULTS

Protein/mRNA ratio and codon usage

Although the phase of gene expression at which selection on codon bias acts is still a matter of debate (1), it is commonly hypothesized that the correlation between mRNA level and codon bias is principally determined at the translation level (2,3). The translational hypothesis assumes that the more frequent the transcript is, the stronger the selective pressure is in favoring codons with more frequent cognate tRNAs. If the efficiency of translation is associated with codon usage, causing the observed correlation between mRNA level and codon bias, it should be reflected in an increase of the protein/mRNA ratio with the transcript level. It has been reported that the protein yield exhibits a weak or no correlation with the transcript level (4,5). Here, we use a high-confidence dataset of protein and mRNA cellular levels of 408 genes (6) to test the hypothesis that the number of protein copies per mRNA molecule is constant across different transcript levels:

$$\frac{[prot]}{[mRNA]} = c,$$

$$\log([prot]) = \log(c) + \log([mRNA]),$$

where [*prot*] and [*mRNA*] are the cellular concentrations of protein and mRNA, respectively, and *c* is a constant. From the above equations, if the protein/mRNA ratio is constant for all levels of mRNA or, in other words, if the correlation between codon usage and tRNA abundance does not affect the efficiency of translation, log([*prot*]) should be a linear function of log([*mRNA*]) with a slope of 1 and an intercept of log([*prot*/*mRNA*]).

We found that the regression line of log[*prot*] on log[*mRNA*] exhibits an intercept of 3.5914 and a slope of 1.111333 (R^2=0.724, $p<0.00001$). The plot of residuals versus fitted values is consistent with no violation of the assumption of linearity. The slope is greater than 1, and the linear regression analysis rejected the null hypothesis H_0: slope ≤ 1 (p-value < 0.0006), indicating that the protein/mRNA ratio significantly rises with the transcript level.

Codon bias within a gene

It has been reported that, within a gene, codon usage bias increases along translational direction (7) and, in bacteria, selective forces favour codons that reduce mRNA folding around the translation start (8). In this study, we analyzed the weight of the contributions of different intragene regions to the whole codon bias. We compare the changes of the relative frequencies of codons with the level of gene expression for different intragenic regions. First, we divided the coding sequence of each gene into three equal parts and compared the codon usage of the three regions at different levels of transcription. We found that the differences in codon usage among the three different regions within a gene are negligible when compared with the strong intergenic variations (Supplementary Figure S6). This difference increases if we reduce the length of the analyzed starting region. However, the intragene variation of codon bias remains much weaker than intergenic differences and is

restricted to a limited number of codons. These results indicate that the major causes of codon bias cannot be found in mechanisms that act differently along the mRNA molecule.

REFERENCES

1. Novoa, E.M. and Ribas de Pouplana, L. (2012) Speeding with control: codon usage, tRNAs, and ribosomes. *Trends in genetics : TIG*, **28**, 574-581.
2. Plotkin, J.B. and Kudla, G. (2011) Synonymous but not the same: the causes and consequences of codon bias. *Nature reviews. Genetics*, **12**, 32-42.
3. Hershberg, R. and Petrov, D.A. (2008) Selection on codon bias. *Annual review of genetics*, **42**, 287-299.
4. Ghaemmaghami, S., Huh, W.K., Bower, K., Howson, R.W., Belle, A., Dephoure, N., O'Shea, E.K. and Weissman, J.S. (2003) Global analysis of protein expression in yeast. *Nature*, **425**, 737-741.
5. Lu, P., Vogel, C., Wang, R., Yao, X. and Marcotte, E.M. (2007) Absolute protein expression profiling estimates the relative contributions of transcriptional and translational regulation. *Nature biotechnology*, **25**, 117-124.
6. Trotta, E. (2011) The 3-base periodicity and codon usage of coding sequences are correlated with gene expression at the level of transcription elongation. *PLoS One*, **6**, e21590.
7. Qin, H., Wu, W.B., Comeron, J.M., Kreitman, M. and Li, W.H. (2004) Intragenic spatial patterns of codon usage bias in prokaryotic and eukaryotic genomes. *Genetics*, **168**, 2245-2260.
8. Bentele, K., Saffert, P., Rauscher, R., Ignatova, Z. and Bluthgen, N. (2013) Efficient translation initiation dictates codon usage at gene start. *Molecular systems biology*, **9**, 675.

SUPPLEMENTARY FIGURES AND TABLES:

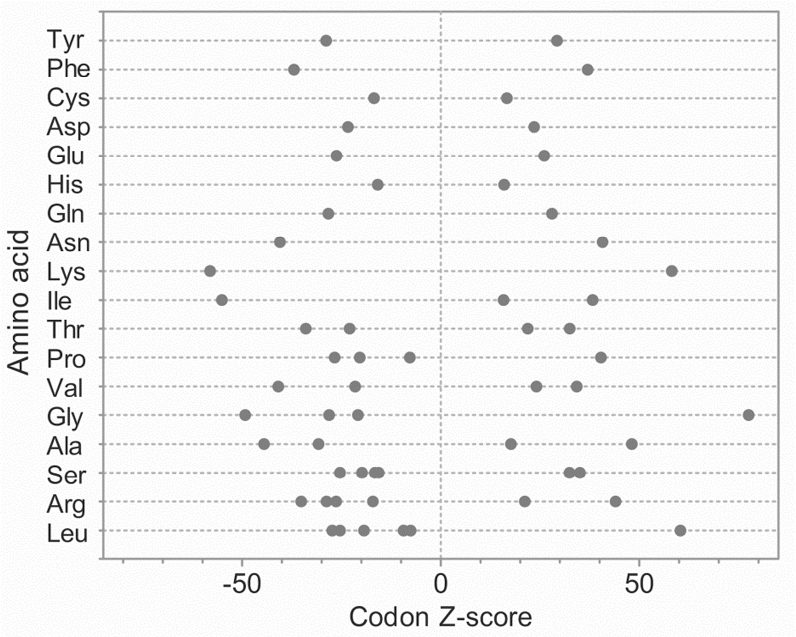

Supplementary Figure S1. Z-score of the average transcript level of native codons with respect to their distribution in a simulation model that mimics the absence of gene-dependent codon bias.

We simulated 5000 genomes with the codon probability at each synonymous site equal to the corresponding fraction in the whole genome. Z-score of the average transcription level of each real codon was calculated on the basis of the distributions of the average transcription level of codons in simulated sequences. The normality of the distributions was tested using the Shapiro-Wilk normality test and the normal probability plot.

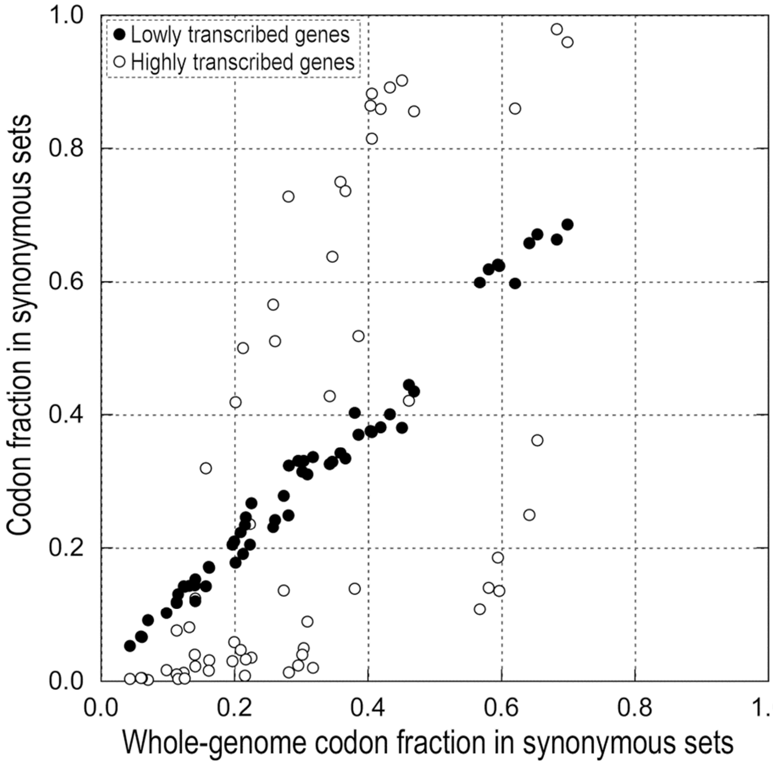

Supplementary Figure S2. Scatter plot of codon fraction in synonymous sets of lowly (closed circles) and highly transcribed (open circles) genes versus codon fraction in the whole genome.

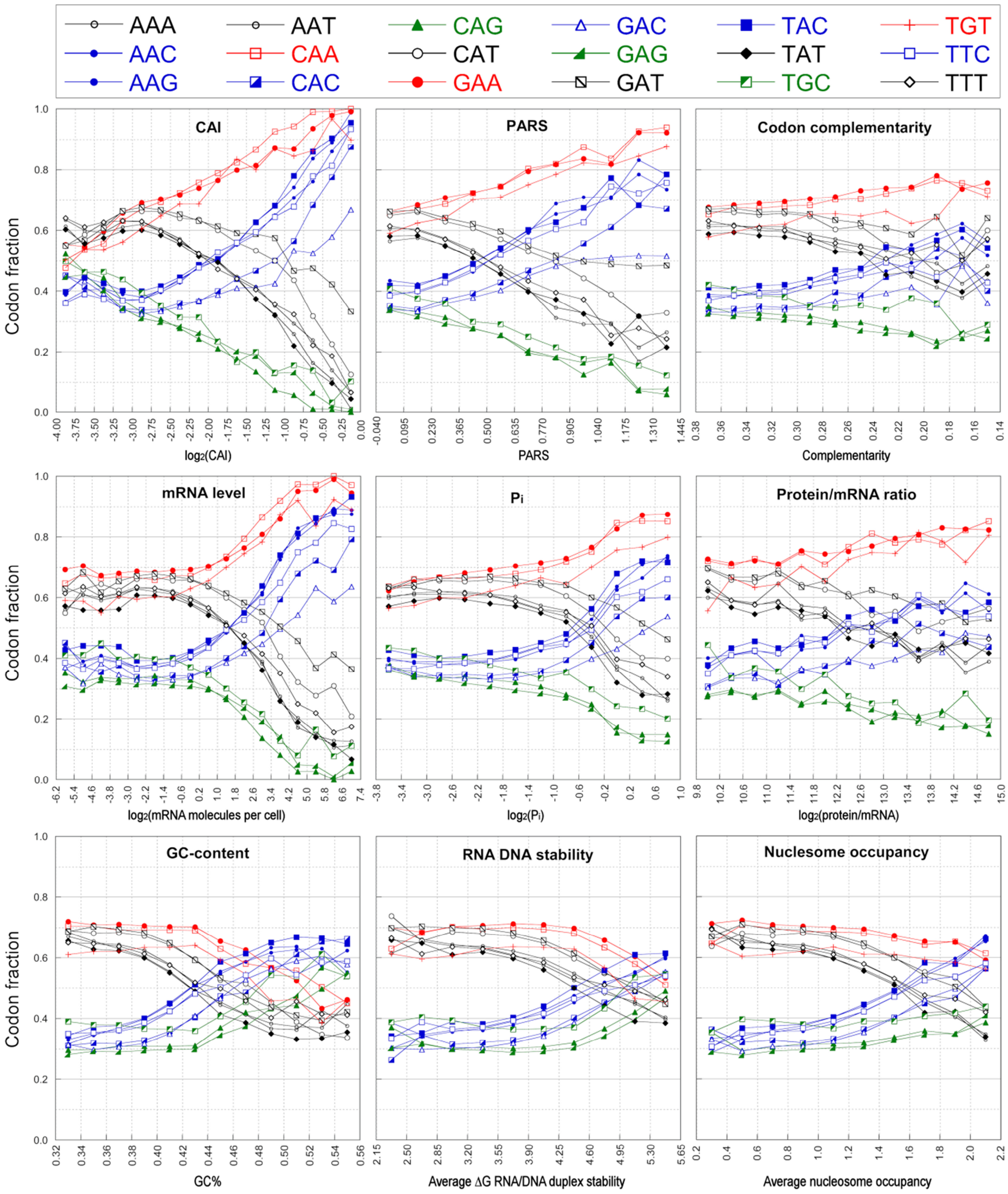

Supplementary Figure S3. Change of synonymous codon fraction of two-fold degenerate amino acids with different coding sequence properties.

Change of major and minor codon fractions with increasing CAI (Codon Adaptation Index) score, PARS (Parallel analysis of RNA structure) score, codon complementarity, level of mRNA, Pi (three-base periodicity index) score, protein/mRNA ratio, GC-content, thermodynamic stability of RNA/DNA and intrinsic nucleosome occupancy. For protein/mRNA ratio we used high-confidence dataset of 408 genes (see Materials and Methods).

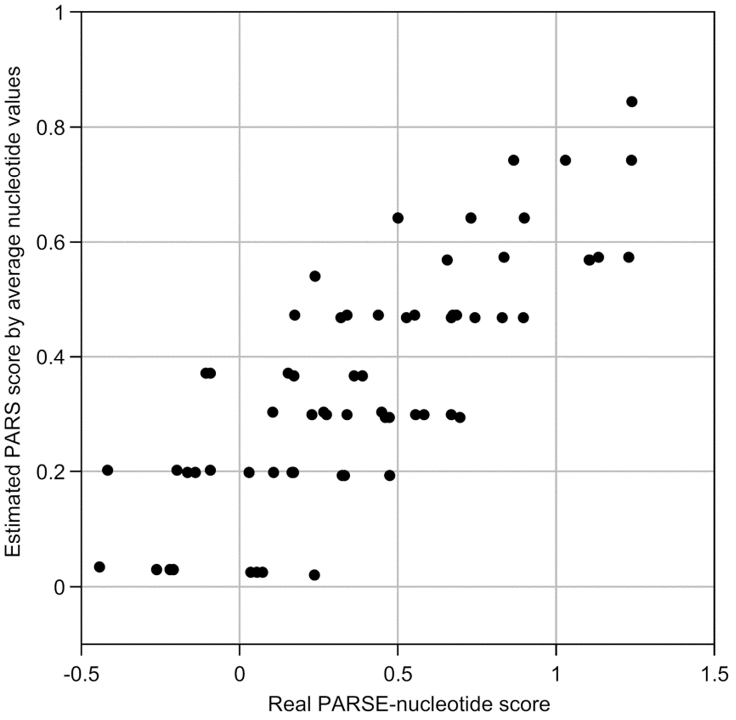

Supplementary Figure S4. Scatter plot of PARS scores of codons estimated by their nucleotide composition versus the average PARSE-nucleotide values.

The estimated values of PARS scores were calculated as the average values of the three nucleotides that compose the codon, where A=0.03, C=0.84, G=0.02 and T=0.54. The PARS-nucleotide score of a codon is the mean of the three values that the experimental PARS dataset associates to its location in the coding sequence.

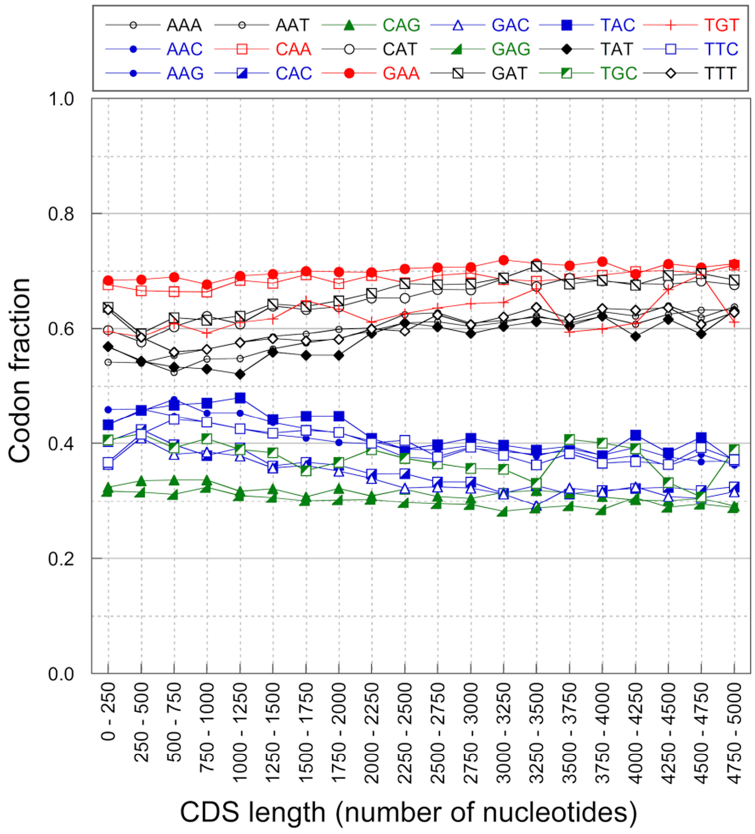

Supplementary Figure S5. Change of synonymous codon fraction of two-fold degenerate amino acids with CDS length.

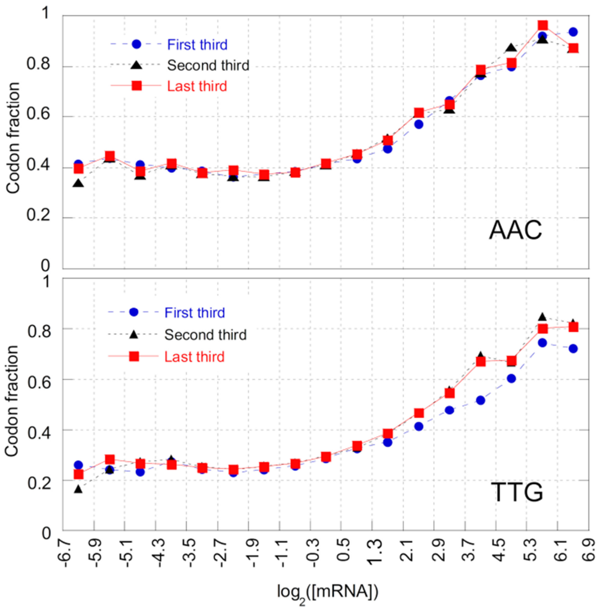

Supplementary Figure S6. Synonymous codon fraction change of AAC and TTG with transcription level.

The coding sequence of genes was divided into three equal parts and the codon usage of the three regions was compared at different levels of transcription. TTG is the codon the shows the highest differences among the codon usage of the three coding regions. The transcript concentration is expressed in molecule per cell.

Supplementary Table S1

Frequency in highly transcribed genes (mRNA per cell>32) and genome-wide PARS-gene score of codons in yeast.

Amino acid	Codon	Frequency	PARS-gene
Lisyne	AAA	185	0.291
	AAG*	1135	0.356
Asparagine	AAC*	563	0.356
	AAT	88	0.286
Glutamine	CAA*	476	0.347
	CAG	10	0.289
Histidine	CAC*	240	0.359
	CAT	80	0.301
Glutamic acid	GAA*	839	0.339
	GAG	35	0.289
Aspartic acid	GAC*	477	0.355
	GAT	271	0.310
Tyrosine	TAC*	355	0.367
	TAT	43	0.296
Cysteine	TGC	17	0.277
	TGT*	105	0.334
Phenylalanine	TTC*	440	0.362
	TTT	100	0.296
Threonine	ACA	51	0.300
	ACC*	513	0.400
	ACG	23	0.287
	ACT*	439	0.376
Proline	CCA*	555	0.377
	CCC	10	0.292
	CCG	8	0.266
	CCT	56	0.316
Alanine	GCA	33	0.304
	GCC	326	0.395
	GCG	5	0.292
	GCT*	1015	0.414
Glycine	GGA	40	0.271
	GGC	66	0.315
	GGG	5	0.286
	GGT*	1023	0.406
Valine	GTA	43	0.274
	GTC*	557	0.401
	GTG	40	0.300
	GTT*	689	0.368
Arginine	AGA*	702	0.345
	AGG	7	0.262
	CGA	2	0.217
	CGC	4	0.274
	CGG	3	0.227
	CGT	102	0.351
Serine	AGC	82	0.296
	AGT	34	0.294
	TCA	50	0.298
	TCC*	345	0.382
	TCG	18	0.292
	TCT*	551	0.370
Leucine	CTA	48	0.304
	CTC	6	0.270
	CTG	13	0.286
	CTT	98	0.282
	TTA	166	0.305
	TTG*	886	0.369
Isoleucine	ATA	11	0.250
	ATC*	459	0.367
	ATT	342	0.331
STOP signals	TAA*	68	0.378
	TAG	6	0.291
	TGA	5	0.273

* Major codons.
